# Body Composition Changes in College Athletes During Holiday Breaks

**DOI:** 10.23937/2469-5718/1510231

**Published:** 2022-08-26

**Authors:** Aston Dommel, R Drew Sayer

**Affiliations:** Department of Nutrition Sciences, University of Alabama at Birmingham, USA

## Abstract

**Background:**

Changes in eating and physical activity during the winter holiday season are commonly associated with weight gain in the general population. Concerns around weight and fat gain are also relevant to collegiate athletes who are generally unable to access on-campus dining and exercise facilities during this time. These concerns were exaggerated in 2020 due to changes in the academic and sports calendar as a result of the COVID-19 pandemic that lead to a holiday break that was 3 weeks longer than normal for many college athletes. The purpose of this study was to investigate changes in Body Mass Index (BMI), Fat Mass Index (FMI) and Muscle Mass Index (MMI) among college athletes during an extended and usual holiday break.

**Methods:**

Fat mass, muscle mass, and weight were measured using bioelectrical impedance analysis as part of routine care in college athletes within two weeks of leaving campus and return to campus during the extended winter break in 2020 (n = 124 athletes) and the usual winter break in 2021 (n = 64 athletes). Change values were calculated for each dependent variable. Differences between extended and normal winter breaks, male and female athletes, and a sex*break interaction were assessed using ANCOVA (BMI and FMI) and Kruskal-Wallis Test (MMI). All analyses were completed using SAS 9.4.

**Results:**

A significant sex*break interaction was observed for BMI and FMI. Male athletes gained BMI and FMI during the extended winter break compared to other sex*break conditions. No differences were found for change in MMI across conditions.

**Conclusions:**

These results demonstrate potential differences in weight and fat mass changes between male and female athletes during an extended holiday break. Future research should investigate whether body composition changes occur during other breaks athletes experience (e.g., summer break) and determine how weight-impacting behaviors such as diet and physical activity differ when they are on campus versus at home. This research can help athletics staff implement strategies to best help athletes maintain optimal body composition and performance during breaks.

## Introduction

In Western societies, the holiday season is a time from November through January where there are many holidays centered around food and celebration. For many years it has been a worry for the general population that there is a tendency to gain weight over the holiday season. Research has shown that there is a significant weight gain over the holiday season, roughly 1kg, due to increase caloric intake and decreased physical activity [1-3]. This weight gain is primarily in the form of fat.

While this amount of weight gain may seem modest, even relatively minor changes in body composition may affect athletic performance in highly-trained athletes. Absolute values of fat mass and fat free mass contribute to the amount of force, power, and speed an athlete is able to produce [4]. Greater fat free mass – skeletal muscle in particular – increases the power producing capacity of athletes, while greater fat mass decreases the efficiency of movement and negatively impacts speed and endurance [5-8]. For most sports, having the highest fat free mass to fat mass ratio is the goal to maximize athletic performance.

In collegiate athletes, body composition has been shown to change over time including within competitive seasons [4], during breaks from structured training [9], and over the course of their college careers [10]. Increases and decreases in both fat free mass and fat mass have been observed. These changes are due to many factors; time of year, type of training, injury, and dietary changes. There is also research showing that athletes may gain weight over the holiday season [11,12]. When athletes have increases in fat mass and decreases in fat free mass performance can decrease [6,13]. Similarly, when athletes go on breaks from structured training, their performance is decreased upon return [14-17].

COVID-19 changed the entire structure of college athletics in 2020. Most spring seasons were cancelled, and many fall season structures were disrupted and some “fall sports” held competitive seasons in the spring [18]. It also lead to changes in the academic calendar for many schools. During a usual winter break, athletes are off campus for roughly 4-6 weeks. Due to COVID-19, many schools transitioned to online school after the Thanksgiving holiday, making an extended winter break off campus of 7-8 weeks [19]. Many areas around the country placed restrictions on business and activities during the holiday season to help mitigate the spread of the virus.

The holiday season is generally a concern for coaches and staff of college athletes due to possible body composition changes and associated performance decreases. This concerned was magnified during the fall of 2020 due to the longer holiday break, less access to resources than usual holiday breaks, and that most primarily fall sports became spring sports during the 2020-2021 school year. The purpose of this research was to investigate whether the extended holiday break resulted in different body composition changes than a usual holiday break in collegiate athletes and whether these changes differed between male and female athletes. It was hypothesized that there would be decreases in muscle mass and increases in fat mass over the holiday breaks, that these changes would be more pronounced during the extended holiday break, and there would be no sex differences in body composition changes.

## Methods

### Protocol

Body composition data were collected on athletes within two weeks of leaving and returning for winter breaks in 2020 (November 2020 and January 2021) and 2021 (December 2021 and January 2022). Due to changes implemented by the university to prevent the spread of COVID-19, the 2020 winter break was approximately 3 weeks longer than the 2021 winter break. Students left campus the week of Thanksgiving and were not allowed to return to campus until January 2021. All study procedures were reviewed and approved by the University of Alabama at Birmingham (UAB) Institutional Review Board, and athletes provided written informed consent prior to their participation.

### Participants

Data for this study were obtained from student-athletes enrolled at a Southeastern division 1 university (UAB). Body composition assessments are routinely conducted on many student-athletes. For this study, the timing of body composition assessments were adjusted to ensure 2-week windows for research purposes. Athletes eligible for inclusion in this analysis were those with valid body composition measures taken within two weeks before and after the holiday breaks in 2020 and 2021. Athletes participated in one the following sports: baseball, softball, women’s soccer, men’s soccer, court volleyball, and beach volleyball. Student-athletes participating in football, men’s and women’s golf, men’s and women’s tennis, track and field, cross country, rifle, and women’s and men’s basketball were excluded from the study due to not routinely having body composition measured, different dates of measurement, or different methods for measuring body composition compared to the sports included.

### Body composition assessment

Body composition and body weight were measured using Tanita MC-780U (Tanita Corp of America, Inc. Arlington Heights, Illinois, USA) a multi-current 8-mode bioelectrical impedance analysis machine. Outcomes were recorded pre and post winter breaks. Outcomes used for this study include body weight, fat mass, and muscles mass. Athletes were assessed in the morning upon waking in a fasted state with lightweight clothing and with socks and shoes removed.

### Statistical analysis

Descriptive statistics were conducted to obtain mean and standard deviation for quantitative variables. Height was used to calculate BMI (kg body weight/m^2^), muscle mass index (MMI, kg muscle mass/m^2^), and fat mass index (FMI, kg fat mass/m^2^) to account for differences in overall body size among the athletes. Analyses were performed to identify potential confounders for the statistical models using correlation analysis with body composition variables. These confounders were included in the appropriate models as follows: for BMI included age, for FMI included age and MMI, and for MMI included age and FMI. For each break, a change variable was created for BMI, FMI, and MMI. These change variables where then compared via general linear models, ANCOVA’s, to test for the main effects of break, sex and a sex*break interaction on each body composition variable. A repeat variable was created to account for those participants (n = 31) who contributed data to both the 2020 and 2021 winter breaks. This repeat variable was included as a covariate in each model. If a change variable was unable to fit the model, a Kruskal-Wallis nonparametric test was used to assess differences between breaks. Significance was set at p < 0.05 for all outcomes and SAS version 9.4 (Cary, NC) was used for all statistical analyses.

## Results

**Figure 1:**
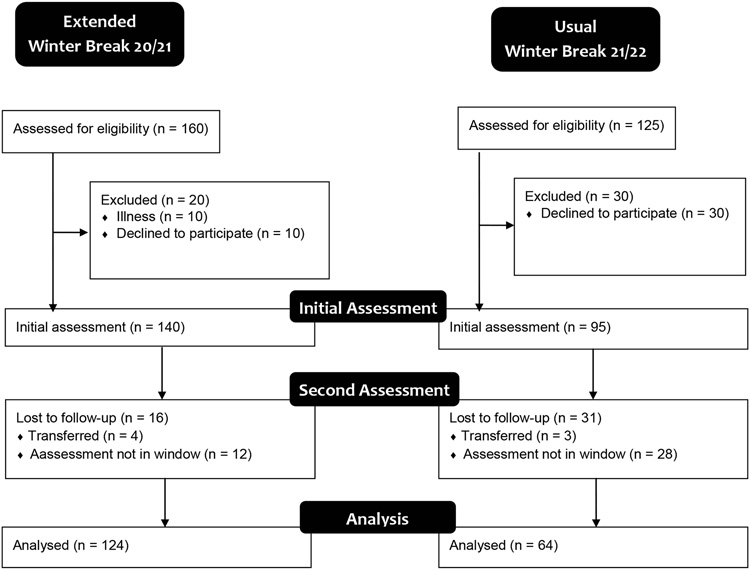
Consort Diagram for extended and usual winter break.

As shown in Figure 1, n = 140 athletes provided informed consent and n = 124 completed both body composition measures during the extended winter break in 2020. Also shown in Figure 1, n = 95 athletes provided informed consent and n = 64 completed both body composition measures during the usual winter break in 2021. Participant characteristics as measured at the pre-winter break time points are shown in Table 1. Ages, weights, and body compositions of the participants are consistent with expectations for college-aged competitive athletes. Table 2 shows body composition change of participants over both breaks.

Table 3 and Table 4 show the results of the ANCOVA for ΔBMI (F = 4.30, p < 0.01). A significant main effect of sex and a sex*break interaction were observed for ΔBMI. Table 5 and Table 6 show the results of the ANCOVA for ΔFMI (F = 4.47, p < 0.01). A significant main effect of sex and a sex*break interaction were observed for ΔFMI. Observed increases in BMI and FMI in males during the extended winter break were greater than changes in BMI and FMI during the usual winter and were also greater than changes observed in females during both winter breaks.

For ΔMMI, the overall models were unable to fit the data. Relationships between ΔMMI and variables of interest were assessed using a Kruskal-Wallis Test nonparametric test and no significant differences were found between ΔMMI and any independent variables.

## Discussion

Results from this study partially support the hypothesis that muscle mass would decrease and fat mass increase, with greater changes seen during the extended winter break. However, muscle mass was not affected by the winter break and sex differences in body composition were not expected. Our research shows that FMI and BMI increased in males during the extended winter break compared to the usual winter break and compared to female athletes during either break. Body composition was generally unchanged among female athletes.

Results of the current study during an extended winter break due to COVID-19 are also largely consistent with previous findings of increased weight and fat mass among college athletes during the initial COVID-19 lockdowns in the spring of 2020 [20,21]. These changes could be due to restrictions placed on gyms and other activities in parts of the country during the winter of 2020/21. Our research is also somewhat consistent with what is seen in the general population during the holiday season, a gain in weight and fat mass in both sexes [1-3]. Our research showed a gain in weight and fat mass in males – but not female – athletes over what is generally considered the holiday season (Thanksgiving – New Year), but only during the extended winter break of 2020. No body composition changes were observed among athletes during the 2021 winter break, which was 3 weeks shorter than in 2020, and the usual length for athletes.

Body composition changes during breaks, both unexpected (e.g.,COVID-19, injuries, family emergencies) and expected (e.g., holiday breaks, summer breaks, postseason) in athletes needs to be better understood to allow athletics staff to best use their time and energy. Breaks are built into athletes’ schedules to allow time for rest and recovery from the strain experienced during intense training and competition. Previous research in athletes has demonstrated that body composition and athletic performance are often affected by breaks from structured training [16,17,22,23]. However, the timing of a break – whether unexpected or expected – in relation to the rest of the season structure or training mesocycle may determine the relative importance of the break on the overall athletic performance. For example, a study in women’s soccer players showed a gain in body fat and loss of muscle mass over the winter offseason, but a body composition had recovered back to baseline prior to the start of the competitive season [11]. In this case, winter is the offseason for soccer, and athletics staff may not be especially concerned with body composition changes during this time because they have the entire preseason cycle to prepare for competition. Alternatively, the winter break for baseball the leads directly into their season, which causes athletics staff and coaches to be more concerned about changes in body composition and performance at this time. The fact that athletes were in different stages of their season could have influenced the results of the current study, due to the variety of sports included and the relation of the winter breaks to their respective competitive seasons. Future research in this field may consider also investigating the timing of breaks from campus residence and training in relation to the athletes’ competitive seasons rather than at specific times in the calendar year as in the current research.

Little is currently known about the differences between the on campus and home environments for athletes that may have driven the results observed in the current study and previous research. For example, access to food, cooking equipment, and food preparation responsibilities are likely different for many student-athletes on-campus compared to at home [24-27]. Athletes in particular are expected to maintain a very busy schedule during school sessions (i.e., classes, team meetings, training, competition) that are much different from a “typical” college student [28-30]. Depending on the resources available at a university (e.g., Power 5 conference vs. smaller universities), athletes have access to a varying degrees of on-campus nutrition support with many larger, well-funded universities providing food and nutrition counseling to student-athletes [31-33]. Athletes may experience different levels of food security at home compared to on campus, 10-25% of athletes were shown to have food insecurity when on-campus, and little is known about how this compares with their home environment [34]. The differences in many nutrition factors on campus compared to home can impact body composition and should be explored further to allow practitioners to better understand how athletes’ food environments interact with changes in body composition and performance in response training and de-training.

A strength of this study is the relative even distribution of sexes with a variety of different sports making results more generalizable across other athletic populations. Body composition assessments were all conducted within two weeks of leaving and returning to campus, which allowed researchers to mitigate the issue of athletes returning at different times. The lack of an objective performance measure in relation to body composition is a limitation of this study that should be included in future research in this population. BIA is both a strength of this study due to its ability to detect both body composition and weight changes and the simplicity of use in an athletic environment that does not typically conduct research. BIA is limited mostly by the influence of hydration, menstruation, exercise, and electrolyte status on measurements. All body composition measurements were collected in a fasted state in the morning upon waking on all occasions to account for these limitations with BIA.

Overall, our study found that weight and fat mass increased in males over the extended holiday break while no body composition changes were observed among the other groups. Combined with previous literature, our results inform practitioners of the impact of breaks from structured training on body composition changes in male and female collegiate athletes. Athletic department staff should be aware of how breaks from campus impact athletes and have protocols in place to best help athletes during these times.

## Figures and Tables

**Table 1:** Descriptive characteristics of athletes as measured prior to the extended winter break in 2020 (n = 124) and usual winter break in 2021 (n = 64). Data are mean ± SD.

	Extended 2020 Winter Break		Usual 2021 Winter Break	
	Male	Female	Male	Female
N	57	67	36	28
AGE (y/o)	20.75 ± 1.50	19.61 ± 1.47	19.92 ± 1.61	20.11 ± 1.81
HT (in)	72.21 ± 2.68	67.82 ± 3.01	72.31 ± 2.71	68.43 ±2.64
BMI	24.85 ± 3.83	23.08 ± 2.71	24.12 ±2.55	23.43 ±2.93
FMI	3.24 ±2.47	5.34 ±2.02	3.06 ± 1.78	5.45 ±2.15
MMI	20.58 ± 1.75	16.83 ± 0.89	20.00 ± 1.10	17.06 ± 1.13

**Table 2:** Results table of body composition change during extended and usual winter break. Data are mean ± SD.

	Extended 2020 Winter Break		Usual Winter Break 2021	
	Male	Female	Male	Female
N	57	67	36	28
ΔBMI	0.42 ± 0.64	−0.06 ± 0.53	0.06 ± 0.64	0.12 ± 0.54
ΔFMI	0.37 ± 0.50	−0.01 ± 0.43	0.03 ± 0.43	0.06 ± 0.46
ΔMMI	0.04 ± 0.46	−0.05 ± 0.28	0.03 ± 0.49	0.05 ± 0.35

**Table 3:** Results of ANCOVA for ΔBMI.

Dependent Variable	Independent Variable	Covariates	F Value	P Value
ΔBMI	Sex*Break	Age	0.01	0.92
		Repeat	0.43	0.51
		Sex	4.85	0.03
		Break	0.56	0.45
		Sex*Break	8.84	0.03

**Table 4:** Results of Tukey Adjusted Means for Sex*Group and ΔBMI.

Sex*Break	ΔBMI Adjusted Mean	95% Confidence Limits	
Male*Extended	0.41	0.24	0.58
Male*Usual	0.06	−0.14	0.25
Female*Extended	−0.07	−0.22	0.07
Female*Usual	0.13	−0.09	0.35

**Table 5:** Results of ANCOVA for ΔFMI.

Dependent Variable	Independent Variable	Covariates	F Value	P Value
ΔFMI	Sex*Break	ΔMMI	1.71	0.19
		Age	0.06	0.81
		Repeat	0.09	0.77
		Sex	5.86	0.02
		Break	2.74	0.10
		Sex*Break	8.63	< 0.01

**Table 6:** Results of Tukey Adjusted Means for Sex*Group and ΔFMI.

Sex*Break	ΔFMI Adjusted Mean	95% Confidence Limits	
Male*Extended	0.37	0.24	0.50
Male*Usual	0.03	−0.12	0.18
Female*Extended	−0.02	−0.14	0.10
Female*Usual	0.07	−0.10	0.24
